# Steady state visual evoked potential (SSVEP) based brain-computer interface (BCI) performance under different perturbations

**DOI:** 10.1371/journal.pone.0191673

**Published:** 2018-01-23

**Authors:** Zafer İşcan, Vadim V. Nikulin

**Affiliations:** 1 Centre for Cognition and Decision Making, National Research University Higher School of Economics, Moscow, Russian Federation; 2 Cognitive Neuroimaging Unit, CEA DRF/Joliot Institute, INSERM, Université Paris-Sud, Université Paris-Saclay, NeuroSpin center, Gif-sur-Yvette, France; 3 Department of Neurology, Max Planck Institute for Human Cognitive and Brain Sciences, Leipzig, Germany; 4 Neurophysics Group, Department of Neurology, Charité-University Medicine Berlin, Campus Benjamin Franklin, Berlin, Germany; School of Psychology, CHINA

## Abstract

Brain-computer interface (BCI) paradigms are usually tested when environmental and biological artifacts are intentionally avoided. In this study, we deliberately introduced different perturbations in order to test the robustness of a steady state visual evoked potential (SSVEP) based BCI. Specifically we investigated to what extent a drop in performance is related to the degraded quality of EEG signals or rather due to increased cognitive load. In the online tasks, subjects focused on one of the four circles and gave feedback on the correctness of the classification under four conditions randomized across subjects: Control (no perturbation), Speaking (counting loudly and repeatedly from one to ten), Thinking (mentally counting repeatedly from one to ten), and Listening (listening to verbal counting from one to ten). Decision tree, Naïve Bayes and K-Nearest Neighbor classifiers were used to evaluate the classification performance using features generated by canonical correlation analysis. During the online condition, Speaking and Thinking decreased moderately the mean classification accuracy compared to Control condition whereas there was no significant difference between Listening and Control conditions across subjects. The performances were sensitive to the classification method and to the perturbation conditions. We have not observed significant artifacts in EEG during perturbations in the frequency range of interest except in theta band. Therefore we concluded that the drop in the performance is likely to have a cognitive origin. During the Listening condition relative alpha power in a broad area including central and temporal regions primarily over the left hemisphere correlated negatively with the performance thus most likely indicating active suppression of the distracting presentation of the playback. This is the first study that systematically evaluates the effects of natural artifacts (i.e. mental, verbal and audio perturbations) on SSVEP-based BCIs. The results can be used to improve individual classification performance taking into account effects of perturbations.

## Introduction

Brain-computer interfaces (BCIs) have potential to help severely disabled people by translating the intentions of subjects into a number of different commands [1]. Due to its safety and high time resolution, electroencephalogram (EEG) based BCIs have become popular and various designs using different signals (e.g. P300 [2,3], sensorimotor rhythms [4,5]) have been proposed. Among them, steady state visual evoked potentials (SSVEPs) are particularly attractive due to high signal to noise ratio (SNR) [6] and robustness [7]. SSVEP is a resonance phenomenon which can be observed mainly in electrodes over the occipital and parietal lobes of brain when a subject looks at a light source flickering at a constant frequency [7]. In this case, there is an increase in the amplitude of the EEG at flickering frequencies and their harmonics and there are different methods to extract the frequency components of SSVEPs. Recently, canonical correlation analysis (CCA) has become a popular approach for analyzing these frequency components as its performance was higher compared to traditionally used Fourier transform [8] and minimum energy combination [9]. Several extensions to standard CCA method were proposed and their performances were evaluated [10].

Robustness of CCA [11] and SSVEPs [12–14] to different experimental conditions were mentioned in several papers. In [11], authors showed that CCA was robust to walking (movement) conditions in SSVEP detection. In [12], authors systematically evaluated the effects of walking locomotion on the SSVEPs using a mobile EEG system and showed that the SSVEP offline detection accuracy decreased as the walking speed increased. In [13], the authors found lesser mental load and fatigue for motion-reversal visual attention task compared to the paradigm with the conventional flickering. In another study, authors showed that an addition of a visual noise can boost both offline and online performances of an evoked potential-based BCI [14]. Although the latter was a steady-state motion visual evoked potential-based BCI, the study is relevant in terms of introducing perturbations. However, in none of the studies mentioned above, there were mental, verbal or audio perturbations introduced to the BCI system. Yet it is exactly these types of perturbations and mental loads that are relevant for the everyday use of BCI outside of the sterile environment of the research laboratories.

In this study, we evaluated a performance of a four-class BCI based on SSVEPs under different perturbations where the subjects were speaking, thinking or listening depending on the given task. We hypothesized that, although the SSVEP is a robust phenomenon, different perturbations (i.e. verbal, mental, audio) should have varying effects within and across subjects due to concurrent performance of another task and thus due to the changes in the attention dedicated to the BCI performance. To the best of our knowledge, this is the first study that systematically analyses the effects of these perturbations on the online performance of SSVEP based BCI within and across subjects.

## Materials and methods

### Participants

Participants were recruited in the summer-autumn 2016 using the database of the Centre for Cognition and Decision Making at Higher School of Economics (HSE). Most of them were students at HSE. There was no payment for the participants and this was made clear before the recruitment. Twenty-seven healthy subjects (eight males) between 18 and 41 years of age (mean: 26 ± 1, SE) participated in the study after giving a written informed consent in accordance with the Declaration of Helsinki. Experiments were approved by the Local Ethics Committee of National Research University Higher School of Economics, Moscow. All subjects participated in both offline and online tasks. However, three subjects (two males) were excluded from the analyses due to their relatively low offline task performances. Two of them reported that they were not able to focus properly in the offline task. Therefore, twenty-four subjects (three left-handed) were included in the results.

### Experiment setup

**Fig 1:**
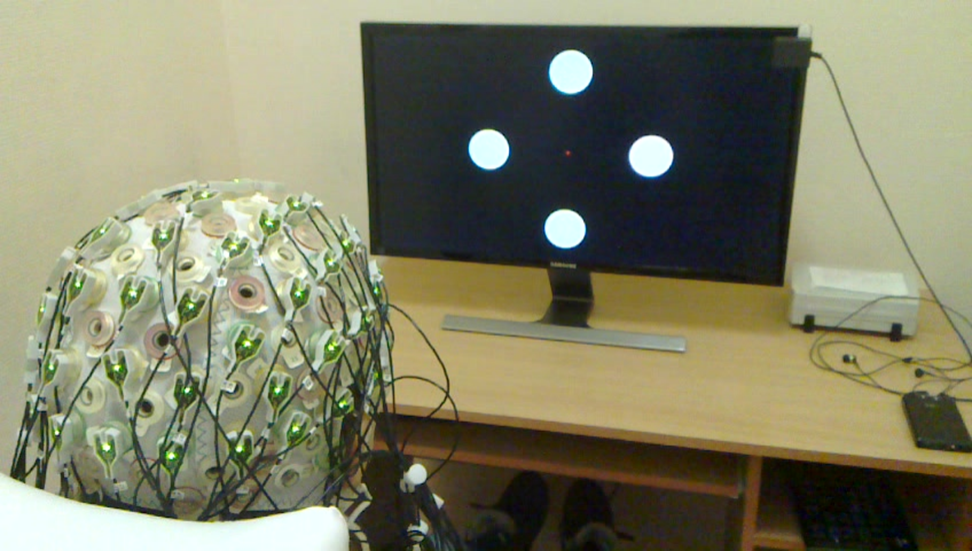
Subject and the position of the flickering circles on LED monitor. Vertical and horizontal field of view were 23.48 and 39.42 degrees respectively. Diameter of the circles corresponded to 3.94 degrees. Circle centers were positioned at 6.92 degrees from the central fixation point.

EEG were recorded in an electrically shielded dark cabin. Stimulus presentation and recording computer was outside of the recording room. Stimulus paradigms were prepared in Matlab software (The MathWorks, Inc., Natick, Massachusetts, United States) using Psychophysics Toolbox Version 3 (http://psychtoolbox.org/). The main stimuli are composed of four circles placed in different locations with individual flickering frequencies (*f*): 5.45 (up), 8.57 (down), 12 (right), and 15 (left) Hz). Participants followed the stimuli presented on a 28 inch Ultra HD LED Monitor (Samsung LED LU28D590DS) with a resolution of 1920 x 1080 pixels and a refresh rate of 60 Hz. Duty cycle was determined as 1/(60/*f*). Hence during one cycle, the related circle was white only in one frame and it was black for the other frames. In this case, when the frame color is reversed once in every four frames, we obtain 15 (i.e. 60/4) Hz. Similarly 12 (60/5), 8.57 (60/7) and 5.45 (60/11) Hz were obtained. In order to avoid performance problems due to the frequency resolution, there was at least 3-Hz gap between the selected frequencies. We did not use frequencies that are multiples of each other in order to prevent the coincidence of the first harmonics of one flicker frequency corresponding to the second harmonics of another stimulus. During the experiment the distance between the participants and the monitor was 90 cm. Fig 1 shows the positions of the flickering circles on the monitor.

### Offline task

**Fig 2:**
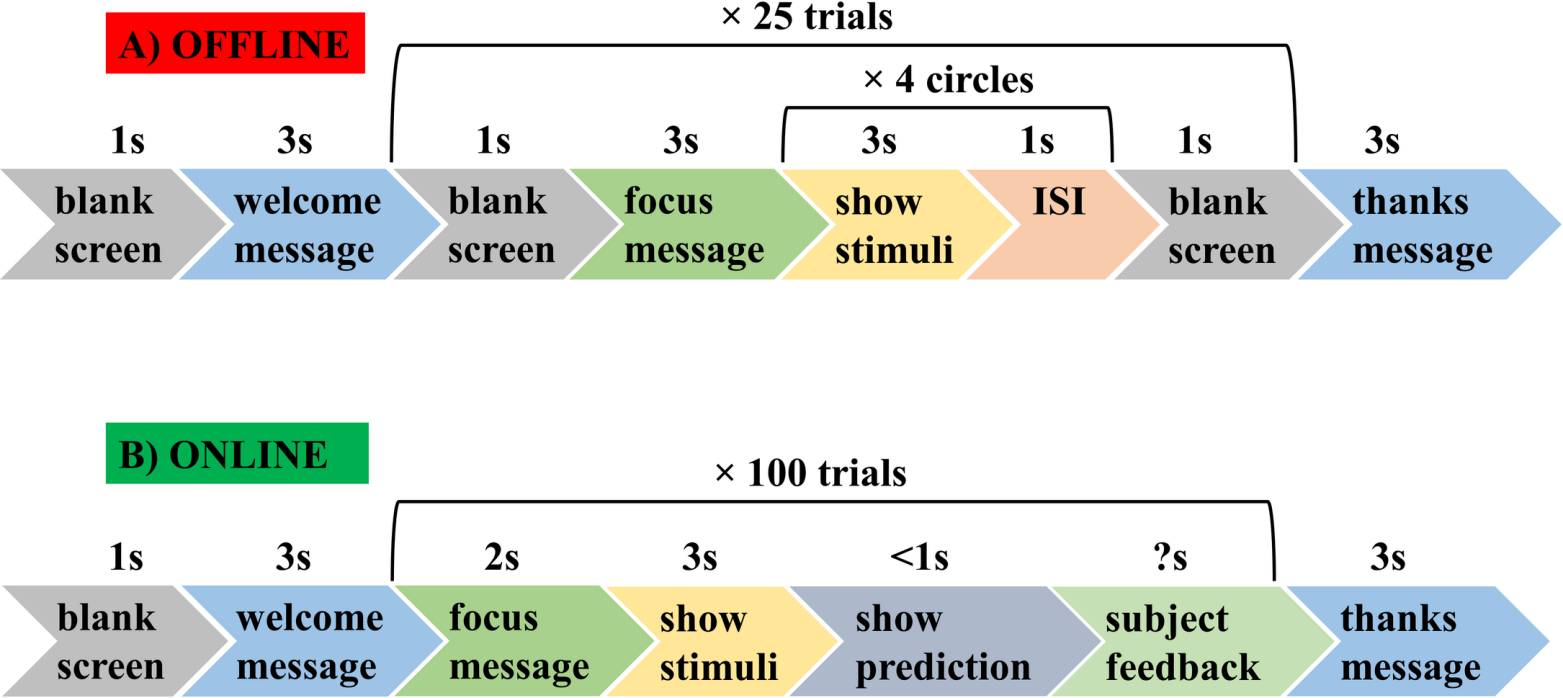
Timing of the tasks. A: Offline, B: Online.

Offline experiment starts with a welcome message after a blank screen. For each of 25 trials, there is an instruction on the screen informing a subject to focus on the presented circle. Subjects then focus on each of the four randomly presented flickering circles indicated by a red oval frame for three seconds with an inter-stimulus interval (ISI) of one second. Trial ends with a blank screen. After all trials are finished, the experiment ends with a "thank you" message. Subjects were instructed to blink during ISI in order to avoid blink-related artifacts during the presentation of the flickering stimuli. This task lasts for about nine minutes. The timing of the offline task is presented in Fig 2A. A demonstration of one trial of the offline task is given in S1 Video.

### Online task

Online experiment starts with a welcome message after a blank screen. For each of 100 trials, there is an instruction on the screen informing subject to focus on the circle. Then, subject focuses on one of the four flickering white circles for three seconds. In this task, the subjects were free to select the circle to focus on. In the end of three seconds, the classification result (i.e. circle in a specific location) is presented on the screen with a red color. Subjects either confirm (pressed ‘Y’ key) or reject (pressed ‘N’ key and then specify the correct response with the arrow keys) the location using keyboard. There is no time limit for the subject feedback. After all trials are finished, the experiment ends with a "thank you" message. The timing of the online task is presented in Fig 2B. A demonstration of one trial of the online task is given in S2 Video.

The online task was performed under four conditions randomized across subjects:

1. Control: There is no counting (no perturbation).
2. Speaking: Subjects counted loudly and repeatedly from 1 to 10 (verbal perturbation).
3. Thinking: Subjects counted repeatedly from 1 to 10 mentally (mental perturbation).
4. Listening: Subjects listened to their pre-recorded voice in waveform audio file format (.wav) with a sampling rate at 22 kHz and sample size of 16 bits) when they were counting from 1 to 10 (audio perturbation).

The subjects counted in a constant speed with which they felt comfortable.

### EEG recordings

EEG were recorded with ActiCHamp amplifier using PyCorder software (Brain Products) from 60 channels using the 64-Channel Standard Electrode Layout for actiCHamp except for FT9, FT10, TP9 and TP10. Reference electrode was on the left mastoid. Three electrooculographic (EOG) electrodes were placed above the nasion and below the outer canthi of the eyes as indicated in [15]. Electrode impedances were kept under 20 kΩ. Sampling rate was 1 kHz.

### Preprocessing

EEG were segmented using the stimuli markers that specify the start and end of the flickering. Trend in the segmented data was removed and the data was filtered with a band-pass filter with cut-off frequencies of 0.53 and 40 Hz in order to remove DC component and high frequency artifacts including power line noise (50 Hz). No extra artifact removal method was used as SSVEPs are not sensitive to low frequency artifacts like eye or body movements [16]. All 60 EEG channels were included in the offline and online tasks to have consistency across subjects. Fieldtrip toolbox was used for both offline and online analysis [17].

### Power spectrum

Fast Fourier Transform was used with Hanning window to calculate the power spectrum of the preprocessed EEG for a 3s stimuli length. The spectrum contained both the first and second harmonics (The n^th^ harmonic = n × the stimulation frequency) of the flickering frequencies. Frequency spectrum included delta (1–3 Hz), theta (4–7 Hz), alpha (8–13 Hz), and beta (14–30 Hz) bands. Relative power was calculated as the ratio of the sum of the power at a specific band (e.g. delta) over the sum of the power in the broad spectrum (1–30 Hz).

### Feature extraction

Canonical correlation analysis (CCA) was used to generate features for classification. For the detailed implementation of the method see [18]. Briefly, we generated data composed of sine and cosine functions that have the same lengths as the segmented EEG data. Then the canonical correlations were calculated between the EEG and the sine and cosine segments. The frequencies of the sine and cosine functions corresponded to the first and second harmonics of the stimulation frequencies. Therefore, two canonical correlation values were calculated for each of the four stimulation frequencies and their second harmonics. These sixteen (8 × 2) correlation values were used as the features for the classification.

### Classification

Decision tree, Naïve Bayes and K-Nearest Neighbor classifiers were used to evaluate the BCI classification performance using features generated by CCA. These classifiers were used in previous SSVEP-based BCI designs in the literature: K-NN [19], Naïve Bayes [20], and Decision Tree [21]. All three classifiers were implemented with the Statistics Toolbox of Matlab. Offline classification accuracy was calculated for different length (1s, 2s, and 3s) of the stimuli using leave-one-out approach. Before the online classification, the classifiers were trained using the whole data in the offline task. All three classifiers were used in the online classification. However, as each classifier generated different classification output, only one of them (Naïve Bayes classifier prediction) was presented to the subject to prevent confusion. Here, the decision to use Naïve Bayes classifier among others was based on its popularity in BCI research. Importantly, when selecting the classifier in advance, we did not know about the final results of classification accuracy among all classifiers, which was only possible to assess upon the completion of all online experiments. Online classification was performed for the stimuli length of 3s.

### Statistical analysis

Two-way repeated measures analysis of variance (ANOVA) was used to investigate the differences in the mean performance of subjects depending on the classifier and stimulus duration factors for the offline task, and depending on the classifier and perturbation factors for the online task. Differences between the classifiers in the offline task and the differences between the conditions in the online task across subjects were evaluated by paired sample t-test with false discovery rate (FDR) correction [22]. Correlations between the relative power during stimuli presentation and the performances across subjects were calculated for delta, theta, alpha (low alpha: 8–10 Hz, high alpha: 10–13 Hz) and beta bands with Pearson correlation coefficient and the significance was estimated using cluster-based permutation statistics, which take into account spatial proximity of the electrodes with significant effects [23]. Differences in the duration of the experiments and power of oscillations in different frequency bands among online conditions across subjects were evaluated using one-way analysis of variance (ANOVA).

## Results

### Offline classification

**Fig 3:**
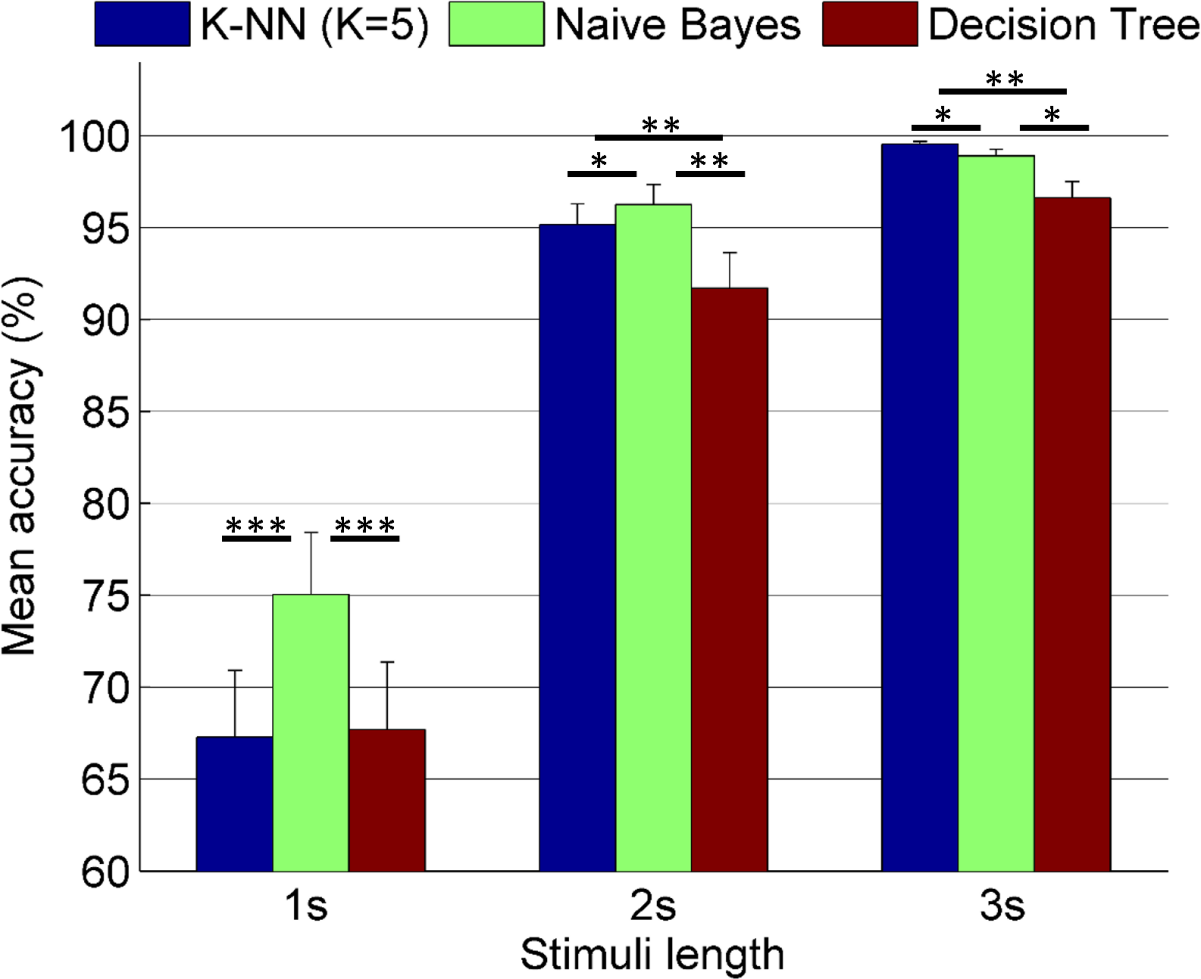
Mean accuracies (%) (N = 24) vs. stimuli length–offline task. *: *p*<0.05, **: *p*<0.01, ***: *p*<0.001 (FDR corrected). Comparisons (t-test) between the classifiers.

In Fig 3, mean accuracies (%) with standard errors are given for varying stimuli length from 1 to 3 seconds in the offline part of the experiment.

In Fig 3, when the stimuli length was short (i.e. 1s), Naïve Bayes classifier accuracy was higher than the accuracy of K-NN and Decision Tree classifiers (p<0.001 in each case). However, there was no significant difference between the performance of K-NN and Decision Tree classifiers. For the larger stimuli length (i.e. 2s), Naïve Bayes classifier accuracy was still higher than the accuracy of K-NN (p<0.05), and Decision Tree (p<0.01) classifiers. For this length, K-NN accuracy was also superior to Decision Tree (p<0.01). For the longest stimuli length (i.e. 3s), K-NN accuracy was higher than the accuracies of Naïve Bayes (p<0.05) and Decision Tree (p<0.01) classifiers. For this length, Naïve Bayes performance was still superior to Decision Tree (p<0.05).

Repeated measures ANOVA (see Table 1) showed significant difference in the mean accuracies in the offline task depending on the classifier, stimuli length and their interaction (*p*<0.001 in each case).

**Table 1:** Two-way repeated measures ANOVA table for mean accuracies in the offline task depending on classifier and stimuli length (i.e. duration).

*Source*	*SS*	*df*	*MS*	*F*	*p*
**Duration**	3.391	2	1.695	82.07	<0.001
**Classifier**	0.081	2	0.040	25.62	<0.001
**Duration x Classifier**	0.049	4	0.012	11.87	<0.001
**Duration x Subject**	0.950	46	0.021	-	-
**Classifier x Subject**	0.073	46	0.002	-	-
**Duration x Classifier x Subject**	0.094	92	0.001	-	-

Mean classification accuracies across subjects were > = 97% for all classifiers (mean ± SD; K-NN: 99.54 ± 0.72, Naïve Bayes: 98.93 ± 1.69, Decision Tree: 96.62 ± 4.30) in offline condition when the stimuli length was 3 s.

**Fig 4:**
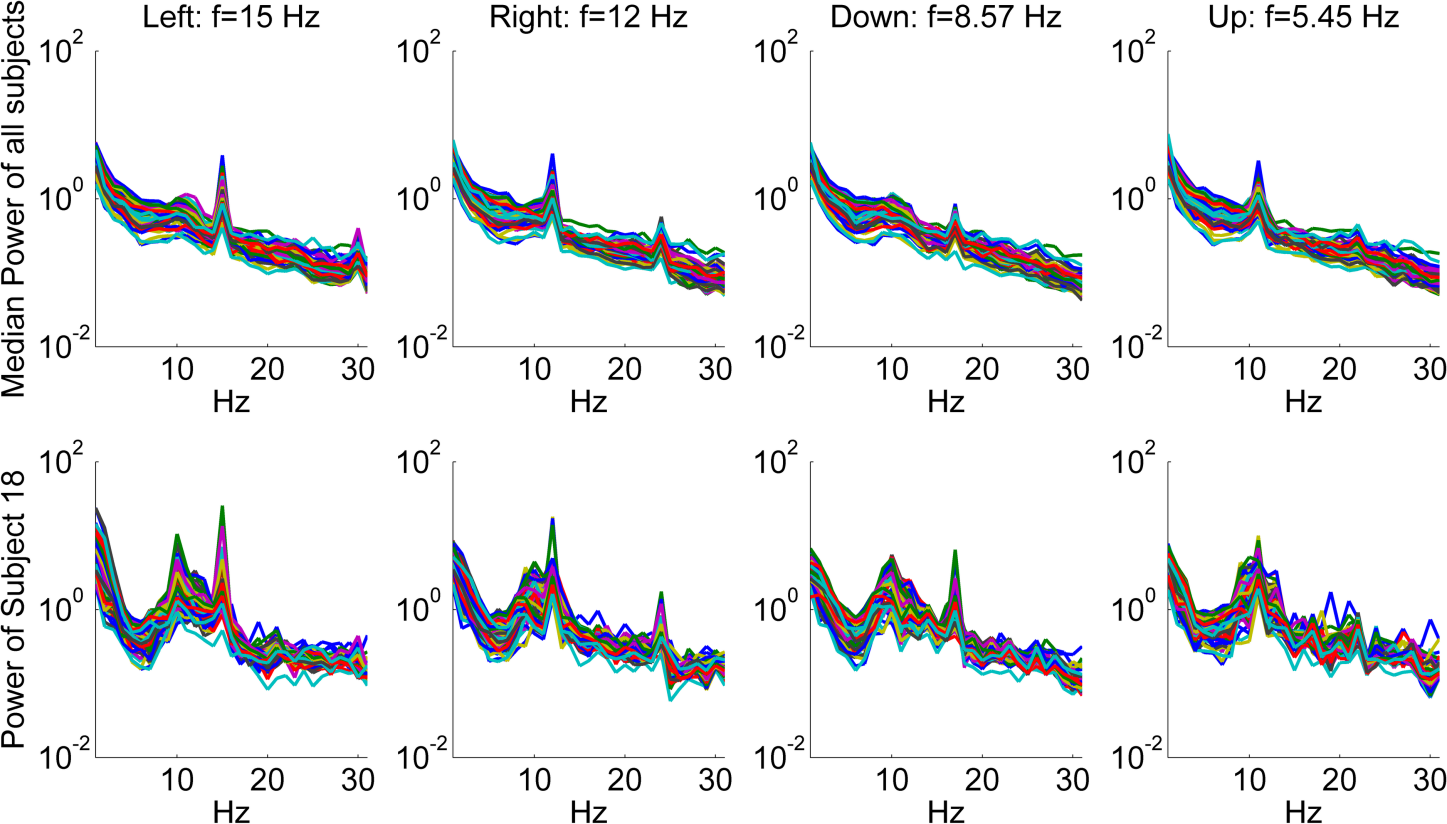
Median power spectrum of all subjects and the power spectrum of a representative subject (Subject 18) depending on the frequency (F) of the circle with attentional focus. Left: f = 15 Hz, Right: f = 12 Hz, Down: f = 8.57 Hz, Up: f = 5.45 Hz. flickering frequency (f) in the offline task. Power was averaged across trials. Each single trace represents one of the 60 EEG channels.

In Fig 4, median power spectrum of all subjects and the power spectrum of a representative subject (Subject 18) are presented depending on the flickering frequency (F) in the offline task.

In Fig 4, peaks corresponding to the first and the second harmonics of the flickering frequencies can be seen. As the alpha band coincides with the first or second harmonics of the flickering frequencies (except F = 15 Hz), some of the stimuli peaks are overlapped with the endogenous alpha oscillations.

**Fig 5:**
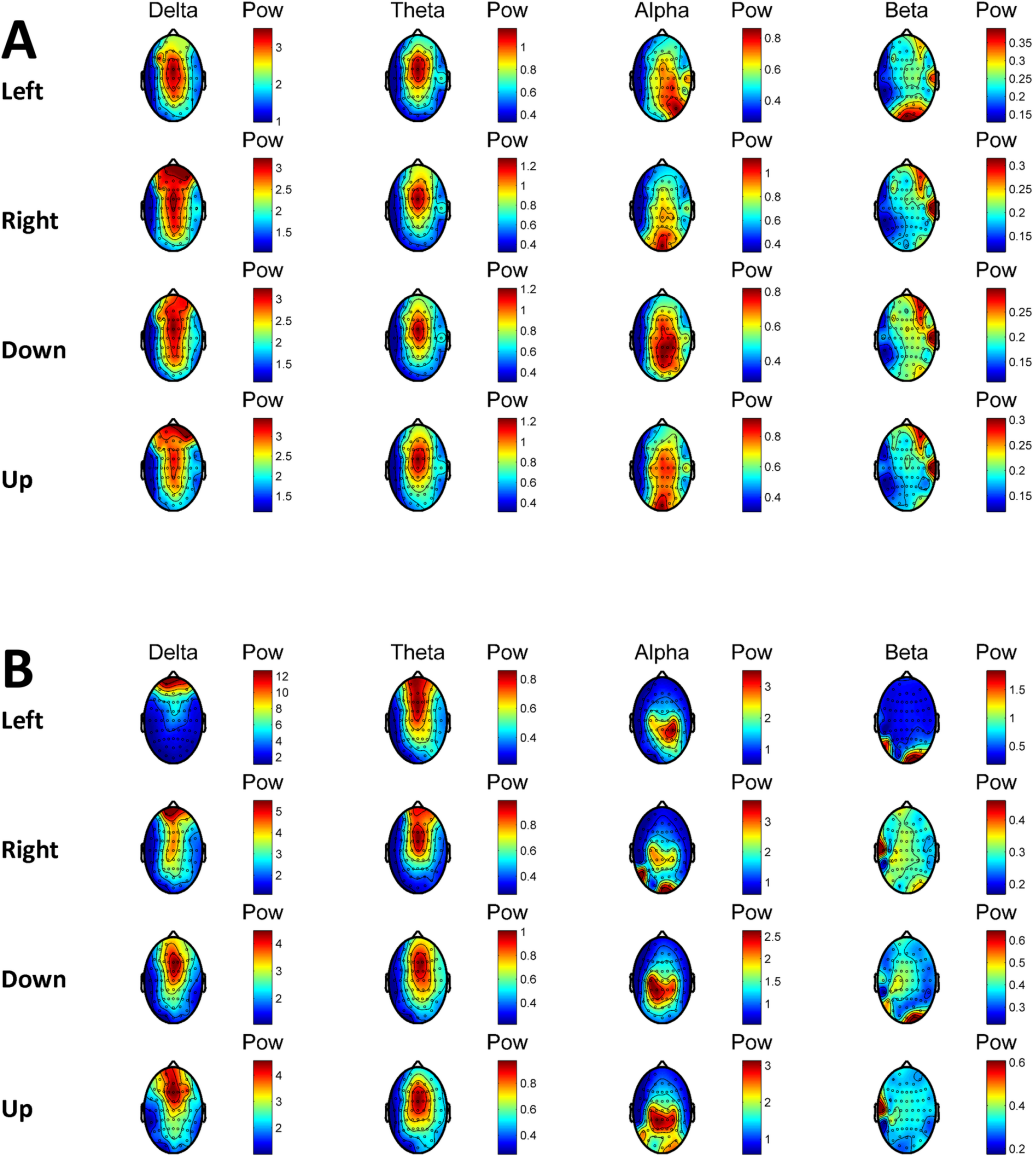
**Median power topography of all subjects (A) and the power topography of a representative subject (Subject 18) (B) in different frequency bands depending on the flicker frequency (F) of the circle with the attention focus in the offline task.** Left: f = 15 Hz, Right: f = 12 Hz, Down: f = 8.57 Hz, Up: f = 5.45 Hz. Power was averaged across trials.

In Fig 5, median power topography of all subjects and the power topography of a representative subject (Subject 18) are presented depending on the flickering frequency (F) in the offline task.

In Fig 5, the increase of the power in the corresponding flickering frequency over the visual cortex can be seen. Besides, the spatial shift of the peak power in the occipital channels depending on focusing to the left or right visual field is noticeable.

### Online classification

**Fig 6:**
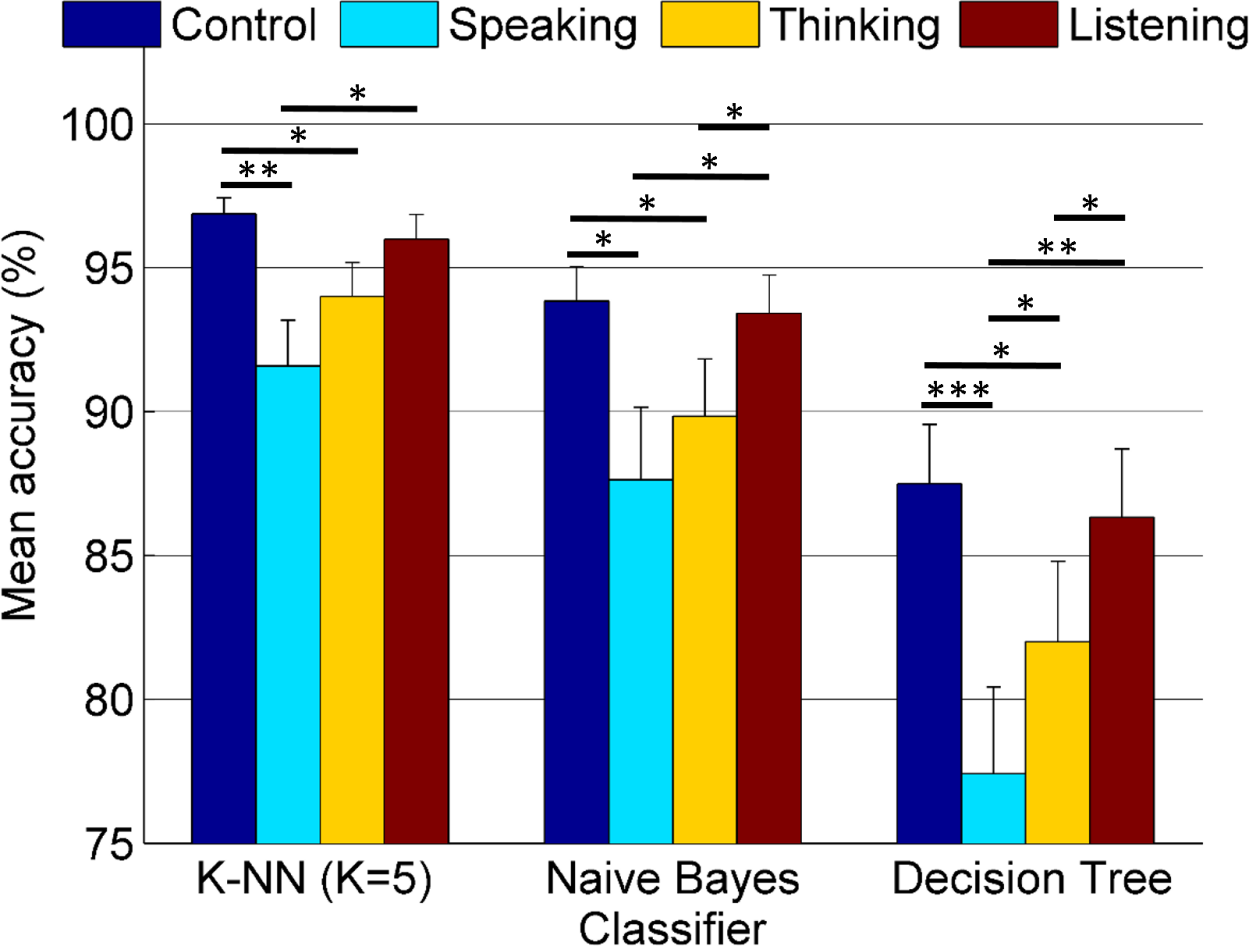
Mean accuracies (%) under different perturbations (N = 24)–online tasks (3 s). *: *p*<0.05, **: *p*<0.01, ***: *p*<0.001 (FDR corrected). Comparisons (t-test) between the perturbations.

In Fig 6, mean accuracies (%) with standard errors are provided for a stimuli length of 3 seconds under different perturbations.

In Fig 6, for the K-NN classifier, accuracy in Control condition was higher than Speaking (p<0.01) and Thinking (p<0.05) conditions. Besides, accuracy in Listening condition was higher than in Speaking (p<0.05) condition. For the Naïve Bayes classifier, accuracies in Control and Listening conditions were higher than in Speaking (p<0.05 in each case) and Thinking (p<0.05 in each case) conditions. For the Decision Tree classifier, again, accuracy in Control condition was higher than in Speaking (p<0.001) and Thinking (p<0.05) conditions. Moreover, performance in Listening condition was also superior to Speaking (p<0.01) and Thinking (p<0.05) conditions. Furthermore, accuracy in Thinking condition was higher (p<0.05) than the accuracy in Speaking condition for this classifier.

Repeated measures ANOVA (see Table 2) showed significant difference in the mean accuracies in the online tasks depending on the classifier, perturbation, and their interaction (*p*<0.001).

**Table 2:** Two-way repeated measures ANOVA table for mean accuracies in the online task depending on classifier and perturbations.

*Source*	*SS*	*df*	*MS*	*F*	*p*
**Perturbation**	2347.8	3	782.6	11.47	<0.001
**Classifier**	6445.0	2	3222.5	24.65	<0.001
**Perturbation x Classifier**	201.7	6	33.6	4.55	<0.001
**Perturbation x Subject**	4707.2	69	68.2	-	-
**Classifier x Subject**	6012.5	46	130.7	-	-
**Perturbation x Classifier x Subject**	1018.7	138	7.3	-	-

**Fig 7:**
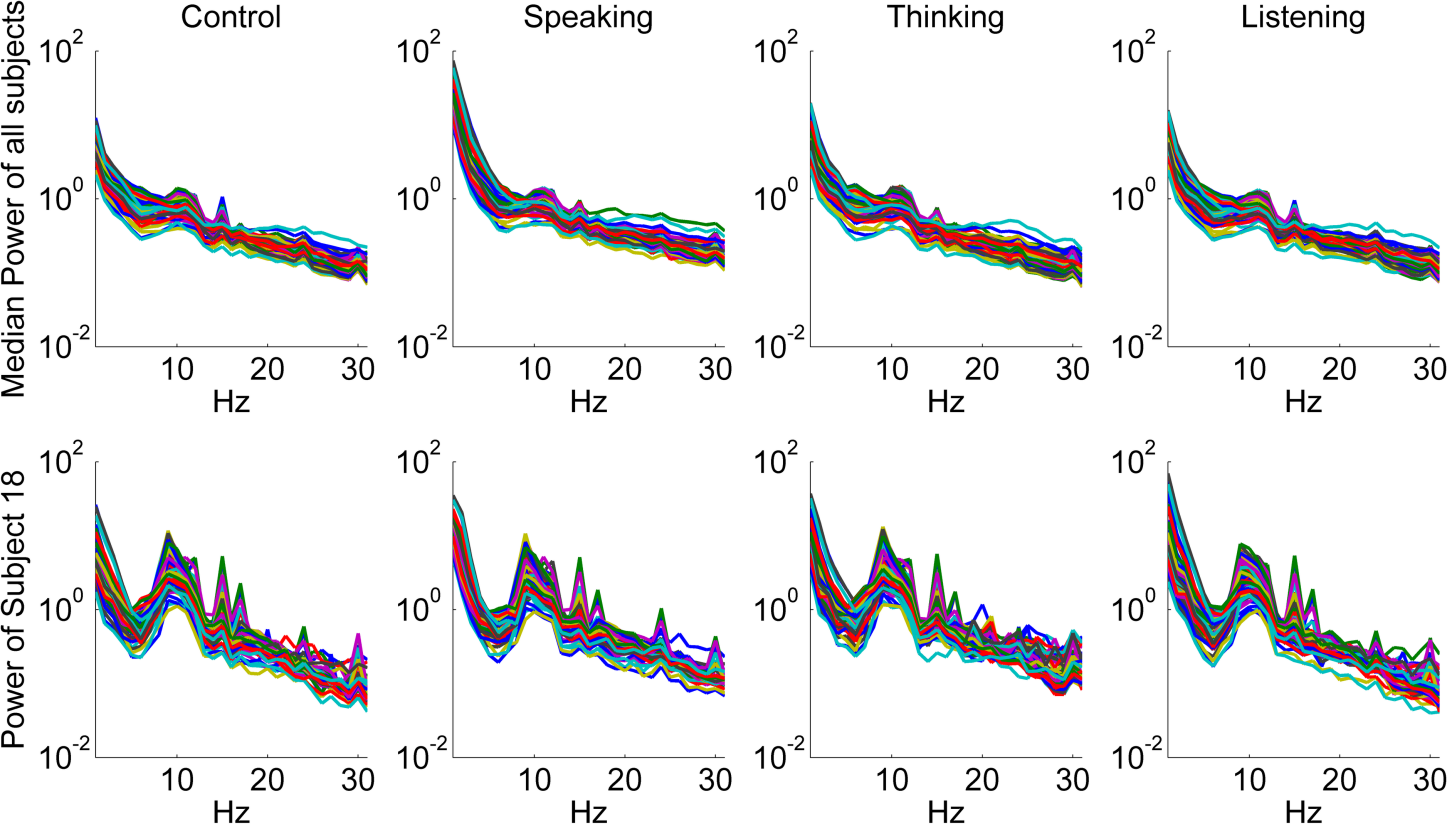
Median power spectrum of all subjects and the power spectrum of a representative subject (Subject 18) depending on the online conditions: Control, Speaking, Thinking, and Listening. Power was averaged across trials. The activity relates to all flickering frequencies. Each trace represents one of the 60 EEG channels.

In Thinking and Speaking conditions during the online task the mean classification accuracy was decreased across subjects in all classifiers compared to the Control condition. However, this decrease was surprisingly small amounting to only about 5%. There was no significant difference between Listening and Control conditions. In Fig 7, median power spectrum of all subjects and a representative subject (Subject 18) were presented depending on the online task using the SSVEP responses from all focused circles.

In Fig 7, median power of all subjects did not reveal pronounced artifacts among the online conditions due to perturbation conditions. Only in the Speaking condition, one could observe that the median delta band power was visibly higher than in the other conditions. To further verify this observation, we averaged power spectra across channels individually for each subject and condition and compared then the power in delta, theta, alpha and beta power bands using ANOVA (See Table 3).

**Table 3:** One-way ANOVA table for power depending on perturbation.

*Frequency band*	*Source*	*SS*	*df*	*MS*	*F*	*p*
**Delta**	**Perturbation**	1084	3	361.332	25.2	<0.001
	**Error**	1146.89	80	14.336		
	**Total**	2230.89	83			
**Theta**	**Perturbation**	3.25	3	1.082	4.5	0.0055
	**Error**	21.17	88	0.241		
	**Total**	24.42	91			
**Alpha**	**Perturbation**	0.23	3	0.078	0.88	0.4533
	**Error**	7.03	80	0.088		
	**Total**	7.26	83			
**Beta**	**Perturbation**	0.06	3	0.021	1.88	0.1405
	**Error**	0.85	76	0.011		
	**Total**	0.92	79			

The results of this comparison showed that the power of oscillations was different only in delta (*p*<0.001) and theta (*p* = 0.005) frequency range. Post-hoc comparisons showed that the power of oscillations in Speaking condition was significantly higher compared to all other conditions (p<0.001 in each case) in delta range and it was significantly higher than in Control (p = 0.015) and Listening (p = 0.009) conditions in theta frequency range. No significant differences at other frequencies were observed. When we restricted the same analysis to Oz electrode, the power of oscillations was different only in delta frequency range (*p*<0.001, F = 16.49) and post-hoc analysis showed that the power of delta oscillations in Speaking condition was significantly higher (p<0.001 in each case) compared to all other conditions in this range.

**Fig 8:**
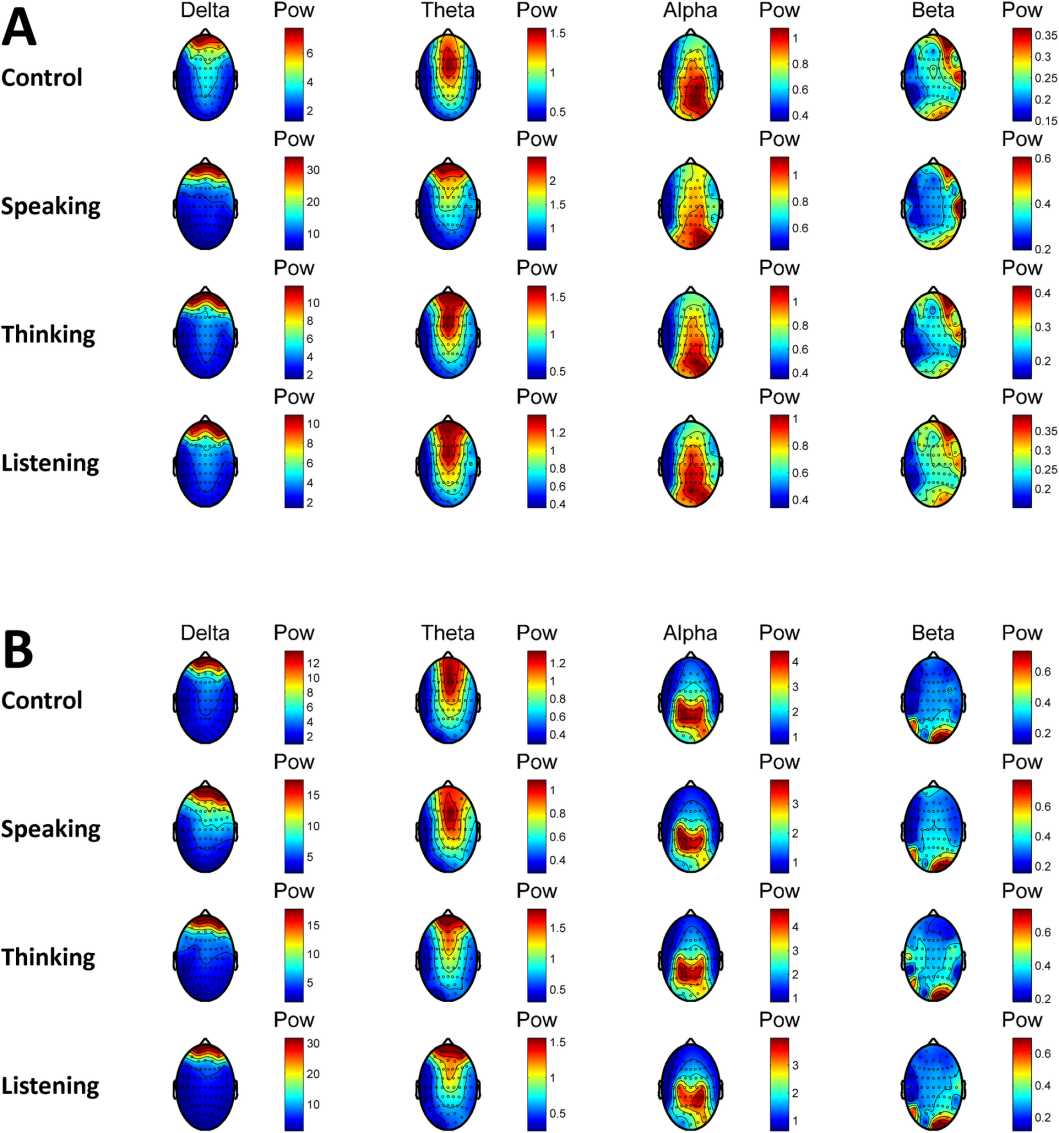
**Median power topography of all subjects (A) and the power topography of a representative subject (Subject 18) in different frequency bands (B) depending on the perturbation condition in the online tasks.** Power was averaged across trials and all target frequencies.

In Fig 8, median power topography of all subjects and the power topography of a typical subject (Subject 18) are presented depending on the perturbation conditions in the online tasks. In agreement with the results presented above, the increase in the delta and theta power in frontal channels can be observed in Speaking condition for the median power of all subjects (A).

For the Naïve Bayes classifier, in Speaking, Thinking, and Listening conditions, the number of subjects with higher performance with respect to the Control condition was 5, 4, and 9 respectively. However, the number of subjects having lower performance with respect to their Control condition was 16, 18 and 12 respectively. The number of subjects who did not show any difference in their performance was 3, 2, and 3 respectively. Among them one subject had 100% accuracy in all conditions.

#### Correlation of alpha power with the performance

**Fig 9:**
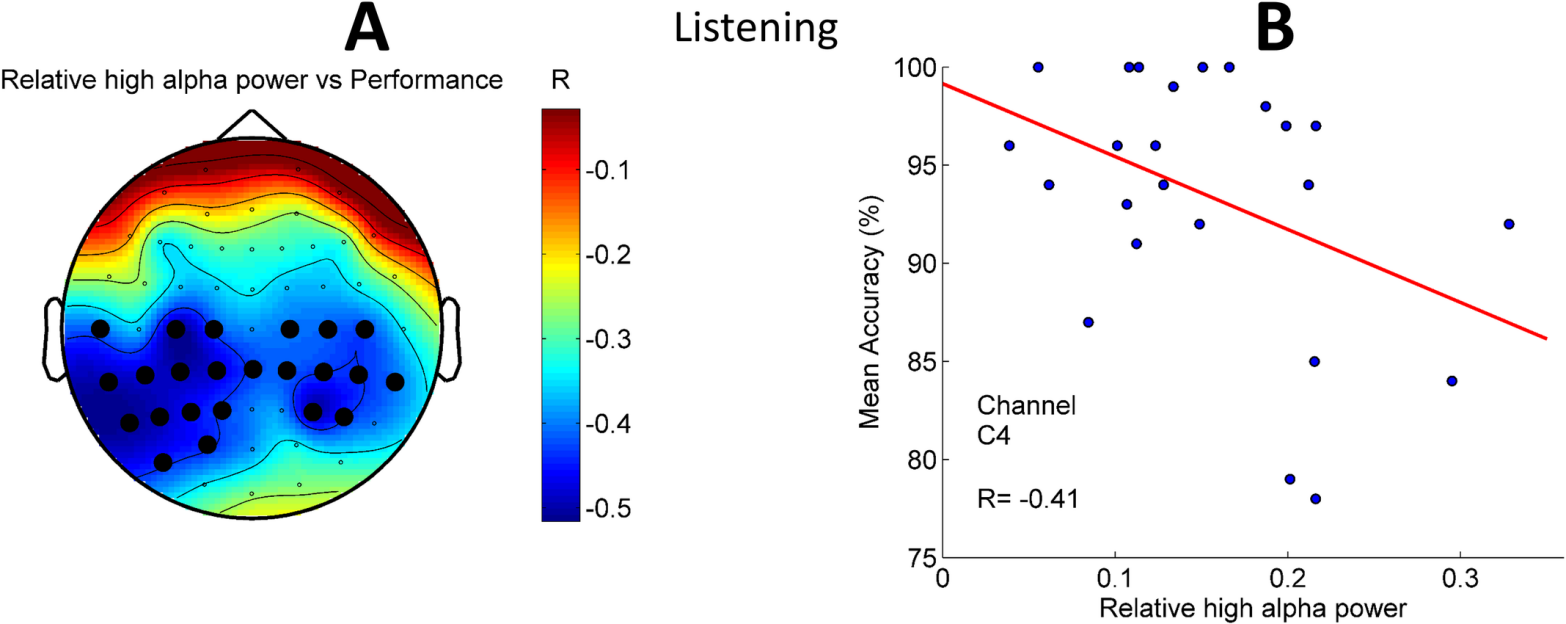
Pearson correlation between relative high alpha (10–13 Hz) power and the Naïve Bayes classifier performance in the Listening condition across subjects. A–Topography of the correlations. Black dots represent the channels that belong to the negative cluster (*p* = 0.02, 1000 permutations). B—An exemplary scatter plot for one of the channels (C4) from the significant cluster.

Alpha oscillations are known to relate to task performance [24,25]. In order to gain further insight into the role of alpha oscillations during the online performance we correlated classification accuracy with the power of alpha oscillations in low (8–10 Hz) and high (10–13 Hz) frequency bands. In Fig 9, a significant negative Pearson correlation (cluster *p* = 0.02) between the relative high alpha (10–13 Hz) power and the Naïve Bayes classifier performance in the Listening task is presented. In none of the other conditions we observed significant correlation between alpha power and the BCI performance.

One-way ANOVA showed no difference in duration of the experiment among online conditions across subjects. The mean duration for each of the online conditions was ~14 min.

## Discussion

To evaluate a dependency of the classifier accuracy, an instance based (K-NN), a probabilistic (Naïve Bayes) and an entropy based (Decision Tree) classifier were used. Different classification approaches can help in capturing the individual differences in brain responses and provide a clue to the understanding of the appropriate classification scheme for each subject.

Interestingly, there was no difference in the duration of the experiments among conditions across subjects. One might expect that the Speaking and the Thinking conditions would last longer as the mean accuracies for these conditions were lower than the Control and the Listening conditions. As subjects should indicate their correct choice in the presence of a mismatch between the classifier prediction and their selection, this potentially might add some extra time to the overall length of the experiment. We assume that subjects were trying to compensate for this loss involuntarily in order to sustain their internal rhythm of counting.

Another interesting observation relates to the individual differences in the evaluation of the difficulty of the conditions. In general, there was no consistency among subjects indicating that one condition was harder than the other, for some of the subjects Speaking was the hardest condition, yet for others Thinking or Listening condition.

Spectral analysis revealed that even during the Speaking condition the spectra of the EEG signals were not strongly contaminated with muscle or motion artifacts in the frequency ranges other than the delta and the theta range. These ranges, however, are usually not used for SSVEP. Because of this we conclude that the decrease in the classification accuracy during the Speaking condition is not only due to decreased quality of the EEG signal in theta band but also due to the other considerations, most likely having a cognitive origin as explained below. This observation is further corroborated by the fact that we also observed a decrease in the classification in the Thinking condition where EEG signals have not demonstrated the presence of artifacts. Silently counting is equivalent to motor imagery which in general activates similar areas as during the performance of real movements [26]. In this sense these two conditions in addition to BCI performance might be considered as an example of a dual task paradigm, which indeed is known to split the attention [27]. This in turn might explain the decrease in BCI accuracy exactly in these two conditions due to the drop in attention relating to focusing on the flickering stimuli. Recently, Brandl et al. [28] investigated the effects of five different distractions in addition to the Control condition on the motor-imagery based BCI performance using Common Spatial Patterns and Regularized Linear Discriminant Analysis. They showed that the decreased performance in distraction conditions can be improved using an ensemble of classifiers and a two-step classification method that first separates the corresponding distraction condition from the other conditions using one-vs-all approach and then applies different classifiers for each group to classify the data into left or right hand motor imagery. The distractions were based on visual (eyes-closed, watching video with a flicker), tactile, muscular and auditory conditions. Although there are similarities between their and our study in terms of the introducing perturbations, there are also considerable differences. Compared to [28] we used SSVEP based BCI. Moreover, we consistently investigated effects of mostly encountered distractors in real world, namely speaking (both loud and internal) and listening. Both studies demonstrate a necessity for further investigation of other perturbations for the development of robust BCI systems.

We did not find a difference in the performance between Listening and Control conditions. If we consider Listening task as a steady-state speech sound condition, this result is consistent with another study, where authors did not find any difference between quiet (i.e. Control in our case) and steady-state speech noise (i.e. Listening in our case) performances in a serial recall task [29].

In addition we also provide a possible implication of our study for the effect of noise on learning processes. Increase in the accuracies under different perturbations for some subjects shows that perturbations should not always be considered as negative in terms of BCI performance. In fact, there is an ongoing debate about the impact of noise and music on the performance in different tasks [30]. It has been shown that white noise improved the working memory performance in a delayed response task in monkeys [31]. However, in a recent study, white noise was detrimental in the working memory task whereas it had beneficial effects in other tasks [32]. Therefore, authors concluded that white noise has differential effects on perception and cognition depending on various factors (e.g. timing of white noise presentation) [32].

Specific to the Listening condition, here we present a neural marker of the task performance based on relative high alpha power in a broad range of cerebral cortex including central, temporal and parietal regions. Subjects with higher relative high alpha power during the stimuli presentation in the Listening condition had lower performance. Interestingly the negative correlation was strongest over the left hemisphere (Fig 9) which is known to be primarily responsible for the language processing [33]. Stronger alpha power usually indicates active inhibition over the areas whose activity should be suppressed [34] for instance in order to avoid effects of task distractors. In our case playback of the counting can actually be considered as a distractor with respect to the performance of the BCI task. Negative correlation between the relative alpha power and performance might indicate that in subjects where the listening to the presented speech had stronger distracting effects (i.e. worse performance)–the suppression was strongest leading to larger power of alpha oscillations over the centro-temporal areas of the left hemisphere. The lack of such correlation in other two distracting conditions (Speaking and Thinking) can also be due to abovementioned presence of two tasks where additional neuronal processing, involved with task switching, cause extra modulation of alpha oscillations thus leading to the masking of the correlation.

We believe that various classification techniques used in our study are sufficient for the demonstration of the effects of perturbations on SSVEP-based BCI performance for the following reasons:

1. As we argue in the discussion, the drop in the performance is likely to be due to the cognitive factors such as divided attention because of the presence of the second task and not due to the degraded signal quality where the use of other classifiers indeed might have an advantage.
2. Although absolute performance might potentially have a boost of a few percent when using other classifiers, the relative effect of perturbations is likely to be similar.
3. The fact that we detected a moderate effect of perturbations using all three classifiers indicates that the detection of perturbations is not related to a specific classifier.

In this study, we limited our feature extraction method to CCA and used CCA-based features with different classifiers. Using lately introduced methods (e.g. multivariate synchronization index [35], temporally local multivariate synchronization index [36]) to detect the frequency in SSVEPs can be a subject of future study.

## Conclusions

We quantified the robustness of a SSVEP-based online BCI under different perturbations using CCA features. Conditions requiring active performance such as loud or silent counting resulted only in slight decrease in BCI performance which indicates that SSVEP-based BCI can be used in parallel during the conversations. The fact that in some subjects perturbations resulted even in better performance indicates that the different cognitive strategy can be used for improving the accuracy of BCI within individuals.

## Supporting information

S1 Video A demonstrative video showing one trial from the offline task. Subject focuses on each of the four randomly presented flickering circles indicated by the red oval frame for three seconds with an inter-stimulus interval (ISI) of one second.
(MP4)

S2 Video A demonstrative video showing several trials from the online task. Subject focuses on one of the four flickering circles for three seconds and confirms (presses ‘Y’ key) or rejects (presses ‘N’ key and then specifies the correct response with the arrow keys) the classification result using keyboard.
(MP4)
